# Interleukin signaling in the regulation of natural killer cells biology in breast cancer

**DOI:** 10.3389/fimmu.2024.1449441

**Published:** 2024-09-24

**Authors:** Jiachi Xu, Hongyu Gao, Muhammad Salman Azhar, Haifan Xu, Siyuan Chen, Mingcan Li, Xinxi Ni, Ting Yan, Hui Zhou, Qian Long, Wenjun Yi

**Affiliations:** ^1^ Department of General Surgery, The Second Xiangya Hospital, Central South University, Changsha, Hunan, China; ^2^ Clinical Research Center For Breast Disease In Hunan Province, Changsha, Hunan, China; ^3^ Breast and Thyroid Surgery, The First Affiliated Hospital of University of South China, Hengyang, Hunan, China

**Keywords:** breast cancer, interleukin, natural killer cell, immunology, immunotherapy, tumor microenvironment

## Abstract

In the field of breast cancer treatment, the immunotherapy involving natural killer (NK) cells is increasingly highlighting its distinct potential and significance. Members of the interleukin (IL) family play pivotal regulatory roles in the growth, differentiation, survival, and apoptosis of NK cells, and are central to their anti-tumor activity. These cytokines enhance the ability of NK cells to recognize and eliminate tumor cells by binding to specific receptors and activating downstream signaling pathways. Furthermore, interleukins do not function in isolation; the synergistic or antagonistic interactions between different interleukins can drive NK cells toward various functional pathways, ultimately leading to diverse outcomes for breast cancer patients. This paper reviews the intricate relationship between NK cells and interleukins, particularly within the breast cancer tumor microenvironment. Additionally, we summarize the latest clinical studies and advancements in NK cell therapy for breast cancer, along with the potential applications of interleukin signaling in these therapies. In conclusion, this article underscores the critical role of NK cells and interleukin signaling in breast cancer treatment, providing valuable insights and a significant reference for future research and clinical practice.

## Introduction

Innate immunity and adaptive immunity represent the core defensive mechanisms of the human immune system. Among these, natural killer (NK) cells—an integral subset of innate lymphoid cells (ILCs)—are crucial for addressing intracellular microbial threats and mediating tumor responses (1). Unlike cytotoxic T cells, NK cells do not necessitate antigen presentation for their cytotoxic functions. This characteristic provides a complementary mechanism to the adaptive immune system (2).

The development, maturation, and cytotoxic functions of NK cells are intricately regulated by various factors, with interleukins (ILs) playing a pivotal role. As cytokines secreted by white blood cells, ILs are essential for mediating cell communication and regulating the immune system. Through their binding to specific receptors, ILs modulate immune cell activity, thereby influencing NK cell growth, differentiation, survival, and apoptosis (3). Each interleukin exhibits distinct biological functions and mechanisms of action within NK cells, highlighting their significant role in immune regulation.

Breast cancer has a profound impact on women’s health globally, being the most prevalent cancer among women in terms of both incidence and mortality (4). Despite significant advancements in breast cancer treatment—such as chemotherapy, radiotherapy, targeted therapy, and surgery—that have markedly improved prognosis (5), a substantial number of patients continue to experience cancer recurrence. Immunotherapy, the latest advancement in treatment, has transformed breast cancer management. Immune checkpoint inhibitors that target CD8+ T cells, particularly PD-1/PD-L1 inhibitors, have significantly expanded and revitalized the tumor-specific T cell pool, leading to notable improvements in patient outcomes (6). However, many patients either do not respond to these therapies or develop acquired resistance. Furthermore, T-cell-based immunotherapies are associated with risks, including excessive T cell proliferation, which can lead to cytokine release syndrome or graft-versus-host disease. In severe instances, these conditions may result in life-threatening attacks by donor cells on the recipient’s tissues (7). These challenges underscore the urgent need for the development of novel immunotherapies that target alternative effector cells.

The clinical success of immune checkpoint inhibitors that stimulate T cells has driven research into immunotherapy strategies targeting NK cells for breast cancer treatment (6, 8). NK cells possess unique attributes not shared by T cells; their ability to simultaneously activate and inhibit multiple receptors helps prevent damage to healthy cells. Furthermore, unlike T cells, NK cells do not undergo extensive proliferation upon activation, which diminishes the risk of cytokine release syndrome or graft-versus-host disease (9). Consequently, many studies have leveraged the tumor-killing capabilities and inherent advantages of NK cells to investigate novel immunotherapeutic approaches for breast cancer (2, 10, 11). In this review, we first summarize the impact of interleukins on NK cell development, maturation, and cytotoxic functions. We then explore the role of interleukins within the breast cancer tumor microenvironment, particularly regarding NK cell-mediated killing of breast cancer cells. Finally, we review the latest clinical studies and advancements in the use of NK cells for breast cancer treatment.

## Development, maturation, and effector functions of human NK cells

**Figure 1:**
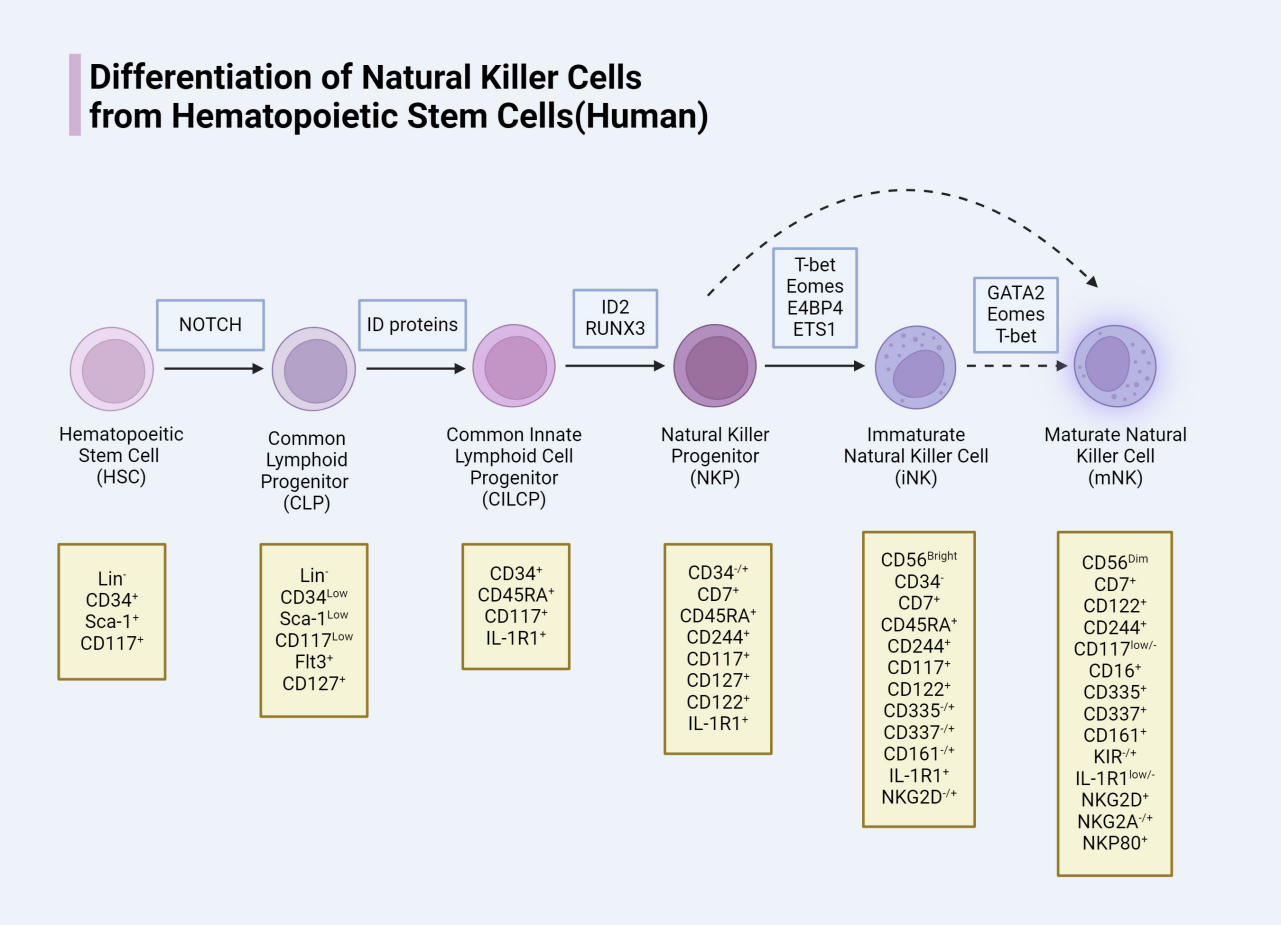
Human NK cells need specific transcription factor, in the process of development and different phases mean a loss of different molecular and expression.

NK cells, belonging to the innate lymphoid cell (ILC) family, constitute 5-20% of all circulating lymphocytes in humans (12, 13). Initially, it was believed that NK cells developed solely in the bone marrow (BM); however, recent studies have revealed that NK cell development also occurs in extramedullary sites, including secondary lymphoid tissues (SLT) such as the tonsils, spleen, lymph nodes, as well as in the liver and uterus (14). In the BM, NK cells originate from multipotent hematopoietic stem cells (HSCs). These HSCs undergo a series of differentiation steps to produce common lymphoid progenitors (CLPs), which further differentiate into common innate lymphoid cell progenitors (CILCPs) with a lineage commitment towards the ILC spectrum. Under the influence of specific transcription factors, CILCPs differentiate into natural killer progenitors (NKPs) with a commitment to the NK cell lineage. Subsequently, cytokines such as T-bet and Eomesodermin drive the maturation of NKPs into functional NK cells, including immature NK cells (iNK) and eventually mature NK cells (mNK) (15–17). Although CD56 expression is a marker of NK cell maturation, full maturation is characterized by a gradual downregulation of CD56. NK cells are classified into CD56_bright_ (iNK) and CD56_dim_ (mNK) subsets, with CD56_bright_ NK cells primarily found in SLT and CD56_dim_ NK cells predominantly circulating in the blood (18). Both subsets express activating receptors NKp46 and NKp80 and produce cytokines; however, only CD56_dim_ NK cells exhibit cytolytic activity. Thus, the downregulation of CD56 during NK cell maturation is closely associated with the acquisition of antitumor cytotoxicity (19, 20). CD56bright NK cells are generally regarded as precursors to CD56_dim_ NK cells, with differentiation marked by an increase in CD94/NKG2C and CD16 expression (21). Nevertheless, some studies suggest that CD56_dim_ NK cells may arise directly from NKPs (22). Throughout the differentiation process from HSCs to NK cells, there is a progressive loss of stemness and changes in the expression of various molecules that define different stages of NK cell development and function. We summarize the developmental process and molecular changes in the differentiation of human HSCs into NK cells (**Figure 1**).

## Interleukin signaling’s impact on NK cells

The expression of cytokines and their respective receptors is crucial for NK cell development (23). Interleukins, a diverse class of cytokines, influence NK cells through various mechanisms. They often work in concert with other cytokines to modulate NK cell regulation, particularly during the differentiation of hematopoietic stem cells (HSCs). In the bone marrow, IL-3 and IL-7 play a role in conjunction with cytokines such as SCF/KL and Flt3L/Flk2 in regulating the differentiation of HSCs into common lymphoid progenitors (CLPs) (23). Additionally, Flt3L or SCF can synergize with IL-15 to significantly enhance NK cell proliferation (24).

Interleukins are crucial for the survival, proliferation, and activation of NK cells. Early research demonstrated that both low-dose continuous infusion and intermittent administration of IL-2 effectively expanded CD56+ NK cells in patients with metastatic cancer (25). Moreover, NK cells cultured with IL-2 or IL-15 exhibit enhanced proliferation, activation, and increased sensitivity to therapeutic drugs (26). A study by Perez et al. confirmed that IL-21 significantly enhanced the cytotoxicity of CD56+ NK cells derived from cord blood/CD34+ cells (27). Additionally, the effects of interleukins on NK cells are often interdependent. Synergistic interactions between certain interleukins can amplify their impact on NK cells. Strengell et al. (28) found that the combined action of IL-21 with IL-15 or IL-18 enhances the production of IFN-γ in both NK cells and T cells, leading to increased cytotoxicity. Similarly, Wendt et al. observed that the combination of IL-2 and IL-21 resulted in more pronounced NK cell proliferation (29). Subsequent studies have identified various signaling cascade molecules downstream of key activation receptors (30).

Interleukin signaling plays a critical role in inducing the differentiation of NK cells. While NK cells generally differentiate from hematopoietic stem cells (HSCs), numerous studies have shown that bone marrow-derived CD34+ cells can be induced by cytokines to differentiate into NK cells *in vitro (* 31). This differentiation process is heavily influenced by interleukin signaling. Ambrosini et al. demonstrated that IL-1β inhibits ILC3 while promoting the maturation of cord blood CD34+ precursor NK cells (32). Although IL-12 and IL-15 are also believed to be involved in this differentiation process, further studies are required to confirm their roles.

**Figure 2:**
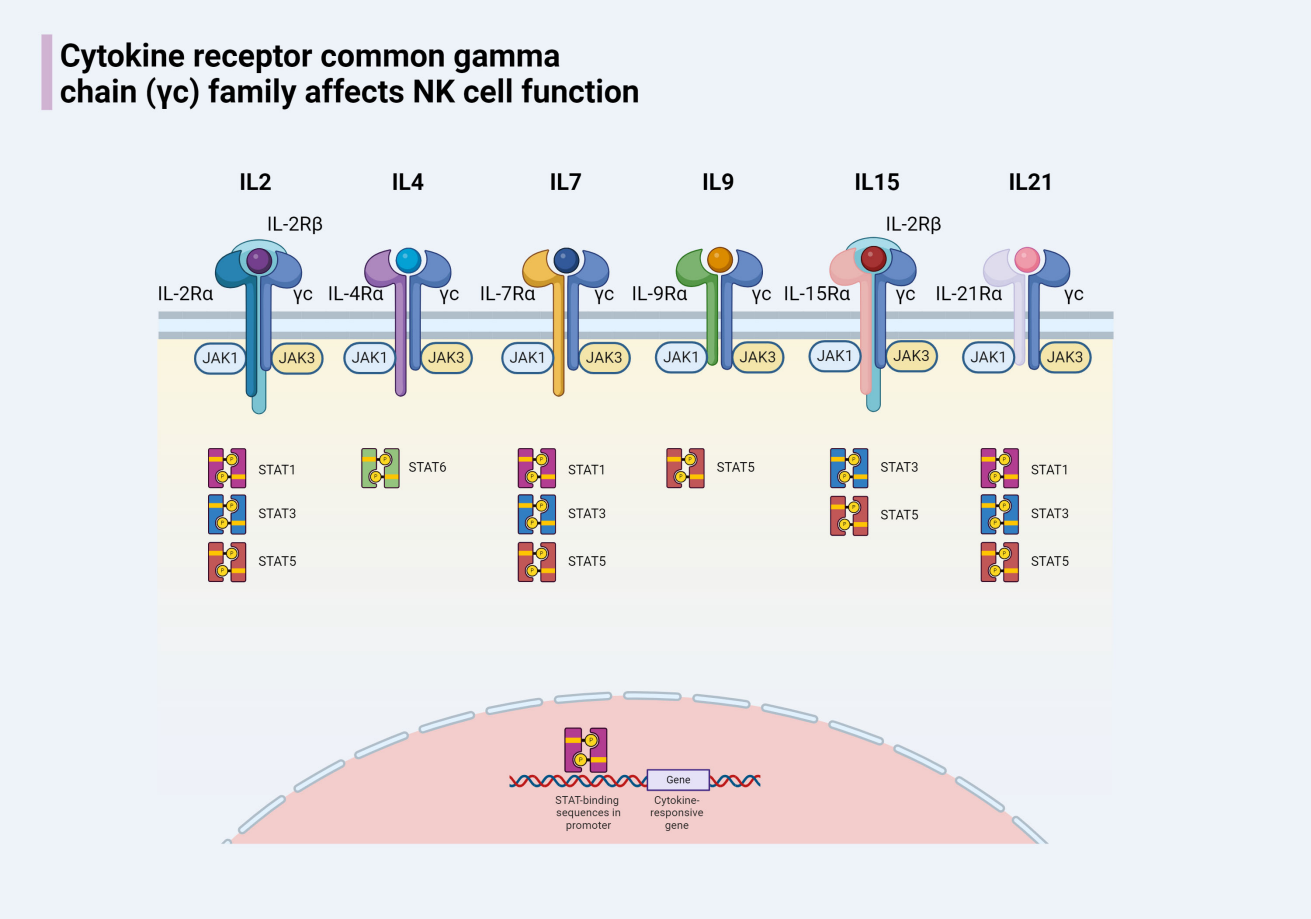
The cytokine receptor common γc chain family of cytokines includes IL-2, IL-4, IL-7, IL-9, IL-15, and IL-21. Members of this family share the common characteristic of having a γc chain and each has a unique α chain. Except for the receptors for IL-2 and IL-15, which have an additional IL-2Rβ chain, the receptors for other members are composed of two chains. When the receptors for members of the γc family on the surface of NK cells bind to their respective cytokines, the associated JAK enzymes are activated, leading to the phosphorylation of STAT proteins. The phosphorylated STATs form dimers and are transported to the nucleus, where they regulate gene expression, affecting the development of NK cells and modulating the immune response.

The common gamma chain (γc) signaling is indispensable for NK cell development, homeostasis, and function. For instance, IL-7 plays a pivotal role in the transformation of HSCs into CD122+ NK progenitors (NKPs), while IL-15 is crucial for NK cell lineage commitment and the maturation of CD122+ NKPs into mature NK cells (33). The γc chain (CD132) is a 40 kDa Type I transmembrane glycoprotein that acts as a shared subunit in the receptors for several interleukins, including IL-2, IL-4, IL-7, IL-9, IL-15, and IL-21 (34). These subunits pair with specific alpha chains to form complete cytokine receptors. Upon cytokine binding, the γc chain is instrumental in activating multiple signaling pathways, particularly the Janus kinase (JAK)/signal transducer and activator of transcription (STAT) pathways, which are crucial for regulating NK cell functions (35). Essential members of the γc family and the key downstream molecules of the JAK/STAT pathway are outlined for a comprehensive understanding (**Figure 2**).

The expression of some interleukin receptors represents different stages of NK cell development. Such as the expression of IL-7Rα (CD127) means that the formation of the CLP, the expression of IL-2Rβ (CD122) means that the formation of NKP, the expression of IL-1R1 is Pre-NK cells into end-stage, will grow into iNK mark. These close relationships constitute the basic architecture between NK cells and interleukin signaling, and interventions targeting the relevant mechanisms are expected to change the status quo of NK cell antitumor resistance.

## Interleukins and the anti-tumor function of NK cells

The anti-tumor function of NK cells begins even before the tumor occurs. An 11-year-long study found that individuals with low cytotoxic reactivity of peripheral NK cells have a higher probability of developing cancer (36). Another study pointed out that patients with congenital NK cell deficiencies have an increased incidence of malignant tumors (37). If routine tumor monitoring fails, that is, when an individual develops cancer, NK cells can still continue to exert their anti-tumor functions.

## The “recognition-connection-elimination” trilogy of NK cells

### Recognition of tumor cells

The anti-tumor process of NK cells can be roughly summarized into a “recognition-connection-elimination” trilogy. NK cells can recognize tumor cells through various mechanisms. The first method is missing self recognition. Normal cells express major histocompatibility complex class I (MHC-I) molecules on their surface, which can send “self” signals to NK cells to prevent them from being attacked (38, 39). However, cancer cells often reduce or lose the expression of MHC-I, thus failing to deliver effective inhibitory signals to NK cells, allowing NK cells to recognize and attack these “self-lacking” cells (40). The second method is the balance of activating and inhibitory receptors on NK cells. As we all know, there are a large number of activating and inhibitory receptors on the surface of NK cells, and the function of NK cells depends on the balance of the activation degree of these receptors. Activating receptors can recognize specific molecules on the surface of cancer cells, such as stress-induced molecules or viral proteins. When the signal from the activating receptor exceeds the signal from the inhibitory receptor, NK cells are activated and attack the target cells (40). Some specific receptors on the surface of NK cells, including the immunoglobulin superfamily (such as KIR), C-type lectin family (such as NKG2 receptors), and natural cytotoxicity receptors (NCR) (41, 42), can recognize specific ligands on cancer cells, thus completing the recognition of cancer cells. In addition, the low-affinity IgG Fc receptor (such as CD16) on the surface of NK cells can also mediate the recognition of NK cells by binding to specific IgG antibodies attached to the surface of tumor cell (43). In addition to the above recognition methods, interleukin signals can also participate in the recognition of cancer cells by NK cells by enhancing/reducing the recognition ability of NK cells. These cytokines (such as IL-12, IL-15, and IL-18) are usually released by other immune cells in the tumor microenvironment and can enhance the recognition and killing ability of NK cells to cancer cells (44).

### Formation of immunological synapse

After completing the recognition of tumor cells, an immunological synapse (IS) is formed between NK cells and them to complete the connection process (45, 46). The term IS originates from the synapse in the nervous system and has similar characteristics of cell-to-cell contact and signal transmission (47). The confirmation of cancer cells enables IS to obtain activation signals, and the activation signals form and stabilize IS through the remodeling of the cytoskeleton, leading to the interaction between NK and target cells, thereby playing the role of immune checkpoints and completing the killing of cancer cells (48). Interleukins also play an important role in this process. The WAS protein can promote the branching of filamentous actin (F-actin) and is necessary for the aggregation of F-actin at the NK cell immunological synapse. However, WAS protein-deficient NK cells have been proven to recover function by activating the WASp homolog WAVE2 through IL-2 *in vitro (* 49). In another study, IL-2 could restore the damage of IS that occurred in the treatment of leukemia by allogeneic transplantation of NK cells derived from umbilical cord blood (50).

### Mechanisms of NK cell-mediated killing

The killing of cancer cells by NK cells occurs through two primary mechanisms: direct killing and indirect killing. In the direct killing mechanism, NK cells recognize cancer cells and deliver granzymes and perforin through the immunological synapse (IS). Perforin inserts into the plasma membrane of the target cell, creating pores through which granzymes enter. These granzymes then activate caspases, which promote a cascade involving IL-1β-converting enzyme (ICE) superfamily proteases (51), leading to the formation of apoptotic bodies and, ultimately, the apoptosis of cancer cells (52). ICE, or caspase-1, plays a crucial role in this process by cleaving the precursors of interleukins, such as pro-IL-1β and pro-IL-18, into their mature, biologically active forms, thereby activating their functions (53). Another direct killing mechanism involves the binding of the CD16 receptor on the surface of NK cells to the Fc region of immunoglobulins. This interaction induces phosphorylation within the immunoreceptor tyrosine-based activation motif (ITAM) domains of the high-affinity IgE receptor and CD3ζ, leading to the targeted killing of cells. This mechanism is known as antibody-dependent cellular cytotoxicity (ADCC) (54). Additionally, NK cells can induce apoptosis in target cells by expressing members of the TNF ligand superfamily, such as Fas ligand (FasL, CD95L) or TNFSF10 (TRAIL, CD253), thereby mediating a delayed killing effect (55, 56).

**Figure 3:**
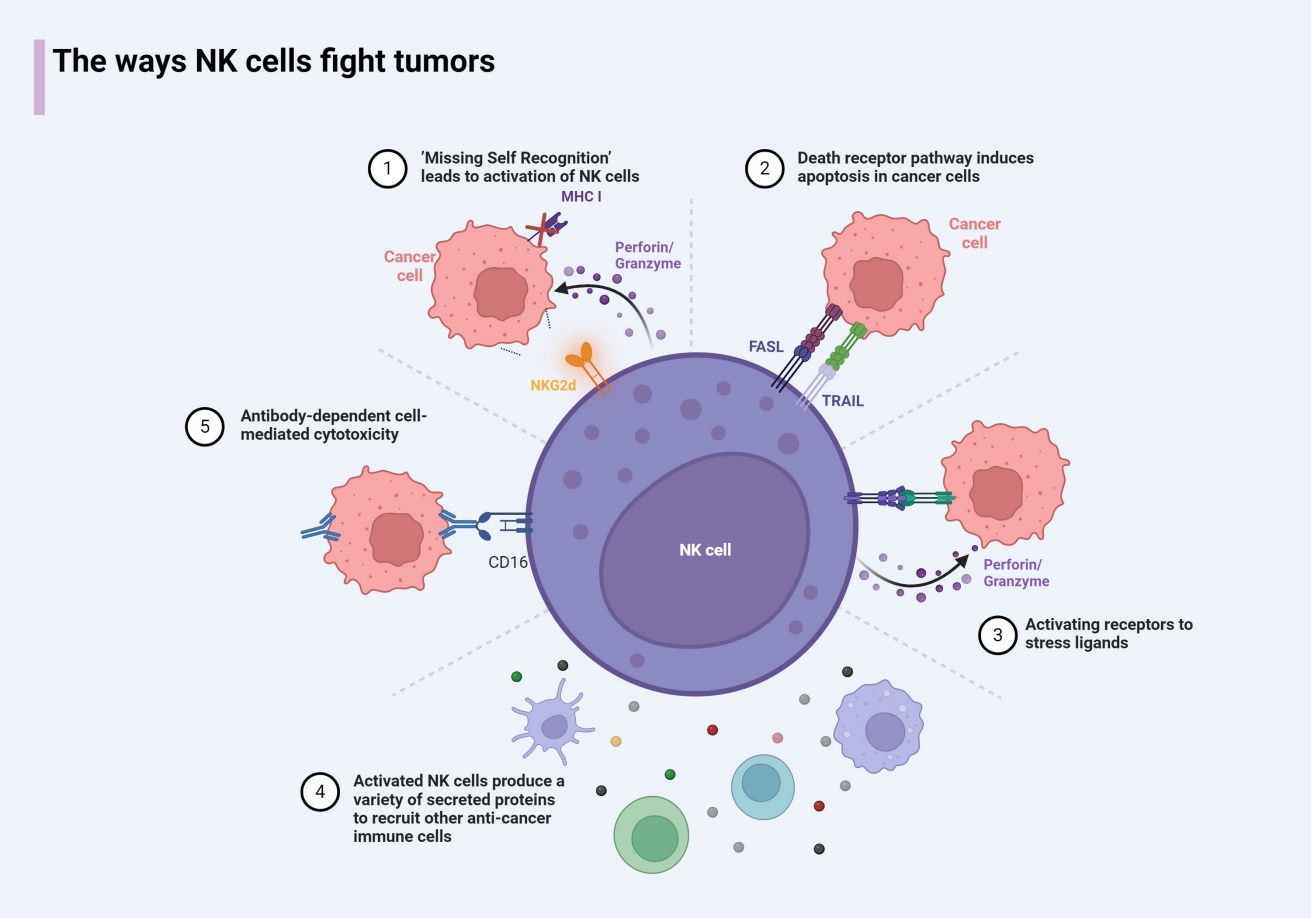
The antitumor mechanism of NK cells. 1) NK cells result in the release of intracellular perforin and granzyme due to loss of inhibition of sensing receptors or upregulation of stress ligands. 2) NK cells express death receptors, which can induce apoptosis in target cells expressing death receptor ligands. 3) Activates receptor that binds to stress ligand, leads to the release of perforin/granzyme by NK cells and completion of killing. 4) NK cells express cytokines that activate the recruitment of other immune cells to the tumor microenvironment. 5) The antibody-bound target activates NK cells through CD16 binding on the Fc portion of the antibody.

NK cells can also complete the killing of cancer cells by promoting other cells to secrete killing substances. When the receptors on the surface of NK cells react with tumor ligands, NK cells will release Th1-type cytokines, including FN-γ, TNF, and granulocyte/macrophage colony-stimulating factor (GM-CSF) (1), activate T cells, dendritic cells (DC), and so on to complete anti-tumor functions (57). We summarize the mechanisms and ways in which NK cells participate in tumor killing (**Figure 3**). In this process, the participation of interleukin signals is also indispensable. Some synergistic effects have produced interesting results, and some immune cells activated by NK cells can also feedback to NK cells. For example, Th1 cells activated by IFN-γ secreted by NK cells can secrete IL-12 to further enhance the activity of NK cells (58). Type I IFN-α/IFN-β triggered by DCs stimulated by NK cells will cause the expression of IL-15Rα on DCs and the production of IL-15 from plasmacytoid DCs (59), which in turn induces the proliferation of NK cells (60). In addition, IL-15, IL-12, IL-23, IL-27, and IL-18 produced by DCs have also had a profound impact on the function of NK cells (61). These findings are crucial for the development of new cancer therapies.

NK cells can also complete the killing of cancer cells by promoting other cells to secrete killing substances. When the receptors on the surface of NK cells react with tumor ligands, NK cells will release Th1-type cytokines, including IFN-γ, TNF, and granulocyte/macrophage colony-stimulating factor (GM-CSF) (1), activate T cells, dendritic cells (DC), and so on to complete anti-tumor functions (57). We summarize the mechanisms and ways in which NK cells participate in tumor killing (**Figure 3**). In this process, the participation of interleukin signals is also indispensable. Some synergistic effects have produced interesting results, and some immune cells activated by NK cells can also feedback to NK cells. For example, Th1 cells activated by IFN-γ secreted by NK cells can secrete IL-12 to further enhance the activity of NK cells (58). Type I IFN-α/IFN-β triggered by DCs stimulated by NK cells cause the expression of IL-15Rα on DCs and the production of IL-15 from plasmacytoid DCs (59), which in turn induces the proliferation of NK cells (60). In addition, IL-15, IL-12, IL-23, IL-27, and IL-18 produced by DCs have also had a profound impact on the function of NK cells (61). These findings are crucial for the development of new cancer therapies.

## Breast cancer tumor microenvironment and interleukin signaling with NK cells

NK cells are known for their significant anti-cancer effects in hematological tumors, but infiltrating and influencing solid tumors, including breast cancer, presents a greater challenge. Nevertheless, current research still associates increased infiltration and activation of NK cells within solid tumors with improved overall survival across various cancers, including breast cancer (62). The infiltration of NK cells into tumors requires them to extravasate from blood vessels and navigate through the extracellular matrix and tumor stroma. While in circulation, NK cells interact with ligands on their surface, such as PSGL-1, via selectin family members like E-selectin, enabling them to roll along the endothelium of blood vessels. During this process, NK cells are activated by chemokines released within the tumor microenvironment, which guide them to tumor sites through the dynamic interaction between chemokine receptors and their corresponding ligands secreted in the tumor microenvironment (TME) (2). Additionally, cytokine signaling within the microenvironment where human NK cells reside significantly influences the type of effector functions they perform (63). However, once NK cells enter the TME, they often encounter negative regulation by immunosuppressive cells and molecules, leading to a functionally impaired state known as “immune exhaustion” (64). To counteract this, it is crucial to dissect the regulatory mechanisms governing the function of tumor-infiltrating NK cells. Moreover, not all tumor-infiltrating NK cells exert anti-cancer effects. For instance, research by Thacker et al. (65) identifies specific immature NK cells that actually promote the progression of triple-negative breast cancer. These diverse findings highlight the complex role of NK cells in the breast cancer microenvironment, with interleukin signaling playing a critical role in these processes. We outline the dual role of interleukin signaling in the breast cancer TME in **Table 1**.

**Table 1:** Interleukin families and their role in breast cancer.

Interleukin	Receptors	Function	Refs
IL-2	IL-2/IL-15Rβ–γc sIL-2Rα IL-2Rα–IL-2/IL-15Rβ–γc	Enhances *in vitro* expansion of NK cells for cytotoxicity against breast cancer cells and IFN-γ production	(66–68)
IL-15	IL-15–IL15Rα + IL-2/IL-15Rβ–γc	Enhances NK cell cytotoxicity against breast cancer cells	(68–71)
IL-21	IL-21R–γc	Enhances NK cell cytotoxicity against breast cancer cells	(72)
IL-8	CXCR1, CXCR2	Increases the sensitivity of breast cancer cells to NK cell-mediated lysis	(73)
IL-10	IL-10Rα–IL-10Rβ	Enhances NK cell activity, impedes breast cancer metastasis	(74)
IL-33	ST2 IL-1RAcP	Enhances NK cell activation and increases the tumor infiltrating NK cells Blocking IL33/ST2 and PD-L/PD-1 enhances the NK cells cytotoxicity of breast cancer cells	(75–77)
IL-30	IL-6Rα-gp130	Inhibits the production of IFNγ by NK cells	(78)
IL22	IL-22Rα1–IL-10Rβ IL-22Rα2	Increases CD155 expression in breast cancer cells, impairing NK cell function by promoting CD226 receptor internalization	(79)

### Carcinostasis

Interleukins and NK cells interact in the immune response, jointly influencing the progression of breast cancer and the outcomes of treatment. Most interleukins can enhance the cytotoxic function of NK cells by activating and expanding them.

#### IL-2

IL-2 is a non-glycosylated protein composed of 133 amino acid residues, with a three-dimensional structure consisting of four α-helices that form a compact bundle. IL-2 exerts its biological effects by binding to its receptor complex, which is composed of three distinct protein subunits: the α, β, and γ chains (80). Upon receptor binding, IL-2 activates signaling molecules such as Janus kinase (JAK) and signal transducer and activator of transcription (STAT). This activation not only promotes T cell proliferation and the generation of cytotoxic T lymphocytes but also enhances the cytotoxic activity of NK cells (81).

As early as 1993, researchers utilized IL-2 to treat peripheral blood lymphocytes (PBLs) from patients with stages I-III breast cancer (n = 41) *in vitro*. The activity of NK cells showed a clear dose-dependent increase and was significantly higher than the baseline NK cell activity observed in healthy controls (66). In the presence of trastuzumab and pertuzumab, IL-2-activated NK cells exhibited significantly higher cytotoxicity levels against SK-BR-3 cells compared to untreated cells (67). Similarly, NK cells stimulated by IL-21 demonstrated significantly increased cytotoxicity against the MCF-7, SKBR3, and T47D breast cancer cell lines (82). Moreover, IL-21 significantly enhanced the cytolytic activity and IFN-γ production of *ex vivo* expanded NK cells when targeting trastuzumab-coated breast cancer cells.

The stability and half-life of IL-2 in the body are relatively short, which limits its efficacy in clinical treatment. To address this limitation, researchers have developed Alb-IL2, a fusion protein composed of human serum albumin (Alb) and IL-2. The design of Alb-IL2 aims to enhance the *in vivo* stability and circulating half-life of IL-2, while reducing potential toxicity, thereby improving its efficacy and safety in clinical applications (83). Studies have demonstrated that the combination of intratumorally injected STING agonists, systemically administered Alb-IL2, and anti-PD-1 checkpoint blockade therapy (collectively referred to as CIP therapy) can stimulate both innate and adaptive anti-tumor immune responses in a triple-negative breast cancer model (83). The synergy between type I interferons activated by STING agonists and IL-2 enhances the expression of IFNAR-1 and CD25 on pulmonary NK cells through a positive feedback mechanism, promoting sustained NK cell activation. These activated NK cells continuously express granzymes, effectively combating and eliminating tumor metastasis.

HER2-positive breast cancer can be targeted by humanized anti-HER2/neu monoclonal antibodies (rhu4D5 or Herceptin). The specific lysis rates of IL-2-activated NK cells against rhu4D5-coated HER2/neu+ (MCF-7Her2/neu) and HER2/neu- (MDA-468) breast cancer cell lines were 35% and 3%, respectively (p < 0.05) (84). In the presence of IL-2, NK cells activated by rhu4D5-coated breast cancer cells also produce substantial amounts of IFN-γ, while surface activation markers CD25 and CD69 are upregulated. Therefore, the concurrent use of rhu4D5 monoclonal antibody and IL-2 treatment in patients with HER2/neu-positive breast cancer may yield superior therapeutic outcomes.

#### IL-15

IL-15 is a multifunctional cytokine composed of 162 amino acids, containing two disulfide bonds and two N-linked glycosylation sites (80). Structurally similar to the IL-2 gene, IL-15 belongs to the four-α-helix bundle cytokine family. IL-15 exerts its effects by binding to the IL-2Rβ/γc heterodimer, which it shares with IL-2, thereby activating the JAK/STAT pathway and promoting the activation and proliferation of immune cells. Various cells, including macrophages, monocytes, dendritic cells, epithelial cells, and fibroblasts, can produce IL-15 (85). This cytokine is particularly crucial for the proliferation and maintenance of NK cells. By binding to the IL-15 receptor on NK cells, IL-15 activates signaling pathways that promote NK cell proliferation and survival. Additionally, IL-15 regulates the terminal maturation of NK cells by modulating the expression of surface molecules such as CD122, which is essential for maintaining NK cell responsiveness to IL-15. IL-15 also enhances NK cell cytotoxicity by upregulating the expression of cytotoxic molecules such as perforin and IFN-γ and acts as a chemotactic factor, aiding in the migration and localization of NK cells. Research by Juliá et al. found that IL-15 enhances the efficacy of trastuzumab in treating triple-negative breast cancer (TNBC) by activating NK cells and dendritic cells (70). The study demonstrated that the combination of IL-15 and trastuzumab promotes the expression of CD25 and CD69 on NK cells and enhances the secretion of IFN-γ and TNF-α, thereby improving the anti-tumor response.

Combined use of recombinant IL-2 and recombinant IL-15 increases the number of NK cells while maintaining their purity (86). In addition, the combined use of IL-2, IL-15, and IL-18 has been shown to upregulate the expression of NK cell receptors, such as CD314, CD158a, and CD107a (69). NK cells stimulated by this cytokine combination secrete higher levels of TNF-α, IFN-γ, perforin (PRF1), and granzyme B, significantly enhancing their cytotoxic activity against breast cancer cells. Studies have demonstrated that IL-2 or IL-15 can restore and augment trastuzumab-triggered antibody-dependent cellular cytotoxicity (ADCC), thereby effectively enhancing the therapeutic efficacy of trastuzumab *in vivo (* 68). The combination of trastuzumab with IL-15 and IL-2 has the potential to improve clinical outcomes in the treatment of triple-negative breast cancer. Furthermore, Roberti et al. demonstrated that IL-2 and IL-15 can enhance trastuzumab-mediated ADCC against breast cancer cells (87). This enhancement is primarily attributed to the ability of IL-2 and IL-15 to upregulate NK cell activation receptors, including CD16 and NKG2D, and to increase the production of IFN-γ.

NK cells release extracellular vesicles (EVs) that carry cytolytic proteins, indicating significant therapeutic potential. Notably, EVs derived from NK cells pre-treated with IL-12, IL-15, and IL-18 have demonstrated the ability to penetrate the core of tumor spheroids. This ability is linked to variations in the expression of NKG2D ligands MICA/B on the surface of tumor spheroids and can be inhibited by the application of anti-NKG2D antibodies (88). Building on this finding, researchers, including Zhu, have enhanced the therapeutic potential of NK-EVs in immunotherapy by pre-treating NK cells with IL-15 (71). They isolated NK-EVs from both untreated NK cells and those treated with IL-15 (NK-EVs^IL-15) from the culture medium of NK-92MI cells. These EVs were then purified using precise ultracentrifugation and density gradient centrifugation techniques. The study revealed that, compared to conventional NK-EVs, NK-EVs^IL-15 exhibited significantly enhanced cytolytic activity against human breast cancer cell lines *in vitro* and promoted the expression of molecules related to NK cell cytotoxicity. In a mouse tumor graft model, NK-EVs^IL-15 significantly inhibited tumor growth without causing substantial toxicity to normal cells or mice, providing compelling evidence for the application of NK-EVs in cancer treatment.

It is worth noting that IL-15 has been shown to delay the formation of primary tumors and prevent or reduce metastasis in various mouse tumor models. For example, when breast tumor cells are intravenously injected into IL-15-deficient (IL-15^-/-^), wild-type C57BL/6, IL-15 transgenic (TG), and IL-15/IL-15Rα-treated C57BL/6 mice, the metastasis rate is significantly higher in IL-15^-/-^ mice, while mice treated with IL-15 TG or IL-15 exhibit almost no metastasis (89). Interestingly, when NK cells are depleted from the control C57BL/6 mice, the number of metastatic foci formed is considerably lower than in IL-15^-/-^ mice, suggesting that in the absence of IL-15, other immune cell types may contribute to metastasis. Further investigation by the authors revealed that the lack of IL-15 in IL-15^-/-^ mice may lead to the polarization of CD4^+ T cells towards a Th2 phenotype, promoting the formation of M2 macrophages, which are believed to contribute to metastasis. This study suggests that while IL-15’s effect on NK cells is crucial, IL-15 also influences other immune cells, which can indirectly contribute to breast cancer metastasis.

Cytokine-inducible SH2-containing protein (CISH) is a key negative regulator of the IL-15 signaling pathway (90). Studies have shown that CISH interacts with JAK1, inhibiting its activity and thereby controlling NK cell responses to IL-15. This regulation is crucial for maintaining NK cell homeostasis and function. Notably, mice deficient in CISH exhibit resistance to breast cancer metastasis, indicating that CISH acts as a potent checkpoint in NK cell-mediated tumor immunity. To further elucidate the mechanisms by which CISH regulates NK cell activity, Bernard and colleagues employed a novel conditional mouse model (Cish^fl/fl^Ncr1^iCre^) (91). Their results suggest that the absence of CISH lowers the activation threshold of NK cells within the IL-15 cytokine signaling pathway. In Cish^fl/fl^Ncr1^iCre^ NK cells, IL-15 stimulation leads to increased expression of IFN-γ and CD107a, thereby inhibiting the metastasis of breast cancer cells.

Early studies demonstrated that recombinant single-chain IL-15 (rhIL-15), produced by E. coli, exhibits significant antitumor activity (91). To further enhance the clinical potential of IL-15, Stravokefalou et al. developed an IL-15/IL-15Rα complex (hetIL-15) to improve its stability and biological activity (92). Compared to rhIL-15, hetIL-15 has a longer half-life and demonstrates superior therapeutic efficacy. The therapeutic effect of hetIL-15, in combination with chemotherapy and surgery, has been investigated in a metastatic triple-negative breast cancer 4T1 mouse model, showing promising results.

Klopotowska et al. found that IL-15 can enhance the tolerance of NK cells to oxidative stress; however, this effect on antioxidant defense is short-lived (93). Additionally, recent studies have indicated that prolonged use of IL-15 may lead to NK cell functional exhaustion (94). These findings highlight the need for further research to determine the optimal use of IL-15 in order to maximize the persistence and expansion of infused NK cells while minimizing the risk of functional exhaustion.

#### IL-21

IL-21 is a member of the IL-10 cytokine family, composed of four α-helices. Unlike some other cytokines that may exist as dimers or multimers, IL-21 typically functions as a monomer (95). Upon binding to its receptor, IL-21 requires a common gamma chain (also known as a common receptor subunit, such as IL-2Rγ or CD132) and a specific alpha chain (such as IL-21R). IL-21 plays a crucial role in regulating immune responses, not only activating NK cells and cytotoxic T lymphocytes but also promoting the differentiation of helper T cells (Th cells) into the Th1 subset (96). Although IL-21 has demonstrated significant therapeutic efficacy in the treatment of various solid tumors, including breast cancer, it faces challenges in clinical application, such as limited response rates and a short half-life. Combining IL-21 with other therapies, particularly PD-1/PD-L1 inhibitors, may further enhance the anti-tumor activity of T cells (97). A recent study explored the combination of apolipoprotein A1-modified doxorubicin liposomes (ApoA1-lip/Dox) with long-acting IL-21 for cancer treatment (72). This combined therapeutic approach not only significantly increased the number of tumor-infiltrating lymphocytes but also enhanced the cytotoxicity of CD8+ T cells and NK cells. The joint application of ApoA1-lip/Dox and IL-21 enhanced the anti-tumor effect while effectively reducing the toxicity associated with ApoA1-lip/Dox, offering a promising new strategy for treating challenging tumors such as triple-negative breast cancer. This approach could potentially improve therapeutic outcomes while reducing adverse reactions, providing patients with a safer and more effective treatment option.

While IL-21 holds considerable therapeutic potential, its use may also induce side effects, such as enhanced inflammatory responses, necessitating careful monitoring and management. The application of IL-21 in breast cancer treatment is still under investigation, and more clinical data are needed to confirm its efficacy and safety. With the rapid advancements in immunotherapy, IL-21 is poised to become an important component in the treatment of breast cancer.

#### IL-8

IL-8 is a chemokine with multiple biological functions that has attracted extensive attention in the field of cancer research in recent years. IL-8 primarily exerts its effects by binding to the CXCR1 and CXCR2 receptors (98). The expression of IL-8 is upregulated in a variety of solid tumors, including TNBC, and it promotes the infiltration of immunosuppressive cells into the tumor microenvironment, angiogenesis, epithelial-mesenchymal transition, and other processes by activating various signaling pathways, such as JAK/STAT and PI3K/Akt (99). These actions further contribute to tumor growth, invasion, metastasis, and drug resistance (100, 101). Studies have indicated that serum IL-8 levels are positively correlated with tumor burden in patients and negatively correlated with the therapeutic effects of immune checkpoint inhibitors. Particularly in patients treated with PD-1/PD-L1 antibodies, higher circulating IL-8 levels are associated with shorter survival times and poorer clinical outcomes. Given the role of IL-8 in tumor development, inhibitors targeting IL-8 are being developed and studied. For instance, HuMax-IL8 is a monoclonal antibody against IL-8 that has demonstrated antitumor activity in preclinical models of low claudin triple-negative breast cancer (73). HuMax-IL8 can not only reduce the mesenchymal characteristics of cancer cells but also decrease the frequency of polymorphonuclear myeloid-derived suppressor cells found at tumor sites. Moreover, HuMax-IL8 increases the sensitivity of breast cancer cells to lysis mediated by immune effector NK cells and antigen-specific T cells *in vitro*. These research advancements suggest that IL-8 plays a significant role in the occurrence, development, metastasis, and immune suppression of tumors, and it may serve as a potential biomarker for predicting therapeutic outcomes and patient prognosis. At the same time, therapeutic strategies targeting IL-8 could offer new options for cancer treatment. Future research is needed to further validate the clinical efficacy of IL-8 inhibitors and to explore the optimal combination with other treatment modalities.

#### IL-10

IL-10 is a low molecular weight cytokine belonging to the four α-helix bundle cytokine family, composed of 162 amino acids. This includes a 48-amino acid signal peptide sequence and a 114-amino acid mature protein portion (102). IL-10 contains two intramolecular disulfide bonds that are crucial for maintaining its three-dimensional structural stability and biological function. Additionally, the IL-10 molecule has two N-linked glycosylation sites, where glycosylation modifications may influence its solubility, half-life, and biological activity (103). When IL-10 binds to its receptor, it first requires high-affinity binding through the IL-10Rα chain, followed by the formation of a high-affinity trimeric receptor complex involving the IL-2Rβ chain and the common gamma chain (γc), which are also shared with IL-2. Upon receptor binding, IL-10 activates signaling pathways such as JAK/STAT, thereby regulating the activity of immune cells. IL-10 is primarily produced by immune cells, including monocytes and macrophages, but it can also be expressed by certain epithelial cells and tumor cells. Studies have shown that IL-10 functions as an effective anti-metastatic agent, particularly in immunocompromised hosts. This anti-metastatic effect appears to be relatively independent of T cell function but is dependent on NK cell activity. Conversely, the inhibitory effect of IL-10 on tumor formation is T cell-dependent (74).

### Carcinogenesis

#### IL-1β

IL-1β is an important inflammatory cytokine that plays a key role in the regulation of immune and inflammatory responses (104). IL-1β promotes the maturation of cord blood CD34+ precursor NK cells (32). In TME, IL-1β is a major cytokine for tumor progression (105). Recent studies have confirmed that IL-1β-induced hypoxia-inducible lipid droplet-associated (HILPDA) mediates the pre-metastatic stage of lung-resident mesenchymal cells (MCs) in breast cancer accumulate neutral lipids. These lipid-laden MCs transport their lipids to tumor cells and NK cells via exosome-like vesicles, leading to enhanced tumor cell survival and proliferation as well as NK cells dysfunction. Furthermore, blockade of IL-1β alone effectively improved the efficacy of immunotherapy using NK cells in attenuating lung metastases (106).

#### IL-22

DNAX accessory molecule-1 (DNAM-1, also known as CD226) is a co-stimulatory adhesion molecule expressed by NK cells and has a crucial role in tumor immunosurveillance (107). NK-92 cells stimulated by anti-CD226 antibody (sNK-92) are more cytotoxic to TNBC cells compared to NK-92 cells (108). As a ligand for DNAM-1, CD155, is expressed on many cell types, including transformed or infected cells, and directly affects NK cell function (109). In breast cancer, for example, high cellular CD155 expression has been associated with aggressiveness and poorer prognosis 31176775. Many kinds of interleukins act on DNAM-1 and further affect NK cell function, such as IL-15/IL-18/IL-27 (110). However, certain interleukins in the tumor microenvironment also inhibit the function of DNAM-1 on NK cells, thus helping tumors to evade immune surveillance.IL-22 acts through the IL-22 receptor (IL-22R) and is associated with a poorer prognosis, higher disease stage, and more rapid tumor progression (79, 111). Briukhovetska and colleagues found that IL-22 produced by T cells can increase the expression of CD155 in breast cancer cells, which in turn disrupts NK cell function by promoting the internalization of the activating receptor CD226. This ultimately leads to the promotion of lung metastasis in breast cancer (79).

#### IL-30

IL-30, also known as IL-27p28 or IL-27A, signals by recruiting gp130 (CD130) to form a homodimer, then uses IL6Rα (CD126) to transmit signals. Among the different molecular subtypes of breast cancer, IL-30 expression is particularly high in triple-negative and HER2-positive subtypes, which may be related to the aggressiveness and poor prognosis of these subtypes. IL-30 can promote the proliferation and invasive and metastatic capabilities of breast cancer cells. Moreover, IL-30 can also provide nourishment for breast cancer stem cells through an autocrine loop of CXCL10 and IL-23, and shape the immune environment and host outcomes, further indicating that IL-30 may play a key role in the maintenance of breast cancer stem cells and the immune escape of tumors (112). Sorrentino et al. found that the lack of endogenous IL-30 triggers the production of IFNγ by T cells and NK cells, thus hindering the progression of triple-negative breast cancer and improving survival (78). However, the specific mechanisms of action of IL-30 in breast cancer and its clinical significance still require further research. Understanding the role of IL-30 in breast cancer may help develop new treatment strategies, especially for aggressive subtypes of breast cancer that currently lack effective treatment methods.

### Two-edged sword

#### IL-6

IL-6 has generally been studied as a derivative of NK cells. In breast cancer patients, tumor-infiltrating NK cell-derived IL-6 is associated with tumors with higher MHC-I expression. In wild-type and IL-6 KO mouse models, inhibition of the IL-6/signal transducer and activator of transcription (STAT3) axis attenuated the suppression of T-cell responses, resulting in reduced tumor growth and metastatic spread (113). Another study showed that anti-IL-6 receptor (IL-6R) ameliorated helper T-cells, cytotoxic T-cells, and NK cells in the lymphatic system cells and reduced Tregs in relapsed and metastatic TNBC. Not only that, the combination of IL-6R and PD-L1 immunotherapy diminished TNBC cell stemness and M2 macrophage activity to a greater extent than monotherapy and showed better survival outcomes and the lowest postoperative recurrence and metastasis rates (114). However, IL-6 is likewise thought to inhibit breast cancer development. A study by Jin et al. demonstrated that NK cells in the TME inhibited the invasiveness of MDA-MB-231 cells, a human TNBC cell line, through IL-6-mediated inhibition of uPA. Cytokine array analysis revealed elevated levels of interleukin IL-10, IL-6, IL-8, C-C motif ligand (CCL)5, and CCL2 in the conditioned medium of co-cultured cells (115). The synergistic effect between these molecules still deserves further exploration.

#### IL-32

It was in IL-2-activated NK cells that IL-32 was originally discovered (116). However, in breast cancer, IL-32-induced vascular endothelial growth factor (VEGF) increases the migratory and invasive capacity of breast cancer and decreases its apoptosis via STAT3 activation (117). Interestingly, it also induces apoptosis and enhances the sensitivity of NK cells and cytotoxic T cells in other cancer types (116). Therefore, we have included it in this review despite the fact that it has not been studied to affect breast cancer after direct action on NK cells.

#### IL-33

IL-33, a member of the IL-1 cytokine family, has a unique fibrinogen-like domain and was identified in 2005 as a ligand for ST2 (also known as IL1RL1) (118). ST2 exists in two forms: a functional long form, ST2L, and a decoy receptor short form, sST2. When IL-33 binds to its receptor ST2, it activates multiple intracellular signaling pathways, including NF-κB and MAPK, which are crucial for the activation, proliferation, and cytokine production of immune cells (119). Despite containing a nuclear localization signal (NLS), IL-33 can also be released as a secretory protein through a non-canonical secretion pathway. IL-33 is primarily expressed by epithelial cells, endothelial cells, and immune cells and can be induced by various stimuli such as injury and inflammation. Recent research has highlighted the role of IL-33 in promoting Th2 immune responses, regulating allergic reactions, and tissue repair (120).

However, the role of IL-33 in tumorigenesis remains controversial. Some studies have indicated that IL-33 can effectively inhibit the development of lung metastases in mouse models of breast cancer. For example, in the 4T1 breast cancer model, IL-33 exerts antitumor effects by promoting macrophages to produce TNF-α and enhancing the activation and specific recruitment of NK cells to the tumor site. The activation of NK cells and the production of CCL5 induced by IL-33 contribute to the recruitment of NK cells in the tumor microenvironment, leading to a potent tumor rejection response (75). Gao et al. found that overexpression of IL-33 in 4T1 cells strongly inhibits tumor growth (121). IL-33 increases the number of tumor-infiltrating NK cells and CD8(+) T cells and their production of IFNγ. The antitumor effect of IL-33 requires the participation of both NK cells and CD8(+) T cells. In contrast, other studies suggest that the IL-33/ST2 axis may accelerate tumor progression and metastasis by promoting tumor angiogenesis and the accumulation of immunosuppressive cells. In breast cancer models, activation of the IL-33/ST2 pathway is associated with accelerated tumor growth and lung and liver metastasis. In tumor-bearing mice, IL-33 treatment reduced the cytotoxicity of NK cells, while the cytotoxicity of CD8(+) T cells did not change significantly, and the removal of CD8(+) T cells had no effect on the progression of breast tumors (76).

Further research has found that in ST2-deficient mice, the inhibition of breast cancer progression and metastasis is associated with enhanced NK cell cytotoxic activity and increased systemic Th1/Th17 cytokines (76). Specifically, there is an increase in the proportion of activated CD27high CD11bhigh NK cells, CD69+ NK cells, and KLRG- NK cells in tumor-bearing ST2-/- mice, as well as enhanced *ex vivo* killing activity of splenocytes, NK cells, and CD8+ T cells. Compared with wild-type mice, there is a significant increase in the number of NK cells expressing IFN-γ in ST2-/- mice, which may be related to an enhanced antitumor immune response.

Additionally, the IL-33/ST2 axis may interact with other immune regulatory pathways, such as the PD-L/PD-1 axis. In a recent study, the authors induced 4T1 breast cancer in BALB/C wild-type and ST2 knockout mice and treated the mice with anti-PD-1 and anti-IL-33. They found that simultaneous blockade of IL-33/ST2 and PD-L/PD-1 delayed tumor onset and slowed tumor growth (77). In ST2 knockout mice treated with anti-PD-1, enhanced cytotoxicity of NK cells against 4T1 tumor cells was associated with overexpression of miRNA-150 and miRNA-155, upregulation of NF-κB and STAT3, increased activation markers, and reduced immune suppressive markers. NK cells in ST2 knockout mice treated with anti-PD-1 tended to proliferate more and were less sensitive to apoptosis. In the spleens and primary tumors of ST2 knockout mice treated with anti-PD-1, the accumulation of immunosuppressive myeloid-derived suppressor cells and regulatory T cells was significantly impaired. Blocking the IL-33/ST2 axis may be a potential tumor treatment strategy. For example, using antibodies against IL-33 or ST2 can inhibit tumor growth and the accumulation of immunosuppressive cells.

Existing research results present differing views on the role of IL-33 in the TME, revealing its complex and multifaceted mechanisms of action. The effects of IL-33 may be influenced by various factors, including the specific TME, immune cells in the tissue, the stage of tumor development, and the type of tumor cells. Future research has the potential to uncover the specific role of IL-33 in tumor immune responses and explore how to develop effective cancer treatment strategies by modulating the IL-33/ST2 axis. In particular, the activation or inhibition of the IL-33/ST2 axis may produce very different immune responses in different tumor microenvironments, which needs to be specially considered when designing targeted therapies. With a deeper understanding of the function of IL-33, we can look forward to new breakthroughs in the field of cancer treatment, especially for tumor types that are not sensitive to traditional treatments.

## Recombinant and engineered interleukins boost function of NK cell to fight breast cancer

Interleukins have demonstrated tremendous potential in the field of breast cancer treatment, particularly with the application of IL-12 family cytokines. However, the full activation of the immune system and the poor stability of interleukins in the body may lead to significant side effects. Therefore, current research trends are focusing on the modification of interleukins or the combination of chemotherapy with immunotherapy, aiming to reduce their toxicity and enhance therapeutic efficacy.

In this context, researchers have designed an innovative nanosystem that utilizes a charge-reversal polydimethylaminoethyl methacrylate (PMet) platform to co-deliver doxorubicin (DOX) and plasmids encoding IL-12. This strategy aims to enhance the treatment effect on metastatic breast cancer through the combined application of chemotherapy and gene therapy. In a 4T1 breast cancer lung metastasis mouse model, this nanosystem can enhance NK cells and tumor-infiltrating cytotoxic T lymphocytes, regulate the polarization from tumor M2 macrophages to activated anti-tumor M1 macrophages, and simultaneously reduce immunosuppressive regulatory T (Treg) cells, increasing the expression of cytokines IL-12, IFN-γ, and TNF-α, showing better anti-tumor and anti-metastatic activity (122).

Furthermore, preoperative intratumoral injection of bio-degradable poly(lactic acid) microspheres (PLAM) loaded with cytokines can stimulate a long-term and systemic tumor-specific immune response. This method utilizes the immune potential of autologous tumors to exert anti-tumor effects with minimal toxicity (123). IL-12, mainly produced by macrophages and dendritic cells, is a heterologous cytokine. IL-12 receptors are expressed on activated CD4+ and CD8+ T cells and CD56+ NK cells; thus, it does not induce the proliferation of resting peripheral T cells or NK cells but has a direct proliferative effect on pre-activated T cells and NK cells. In the Balb/c mouse 4T1 breast cancer model, PLAM injected alone with IL-12 after spontaneous metastasis showed anti-tumor effects. *In vivo* lymphocyte depletion studies confirmed that its anti-tumor effects are mainly mediated by NK cells, but this may enhance the immune suppression of T cells. The combination of IL-12 and TNF-α loaded PLAM as a new adjuvant immunotherapy can enhance the activity of tumor-specific CD8+ T cells. Intratumoral cytokine therapy shows great potential in clinical and immunological aspects, but to produce a lasting tumor-specific T cell response, rather than an NK cell response or a more detrimental immune suppression effect, precise cytokine combinations and sustained delivery methods are needed.

In a recent study, nanoparticles based on the low-temperature expansion effect of acid-sensitive materials mPEG-Dlinkm-PDLLA and Pluronic F127 were used to co-deliver hydrophobic chemotherapeutic drug PTX and the biologic macromolecule IL-12 (124). The nanoparticles enriched in the tumor site significantly inhibited the growth and metastasis of breast cancer cells 4T1 and extended the overall survival time of tumor-bearing mice. The combination of PTX and IL-12 activated T lymphocytes and NK cells to release IFN-γ, selectively inhibited regulatory T cells, induced tumor-associated macrophages to differentiate into the M1 type, thereby improving the tumor immunosuppressive microenvironment.

Gene therapy can well control the location and quantity of interleukins to maximize the improvement of NK cell expansion and function. Zhao et al. injected mice inoculated with 4T1 mouse breast cancer cells with plasmids encoding IL-15 and everolimus and found that both IL-15 gene therapy and everolimus significantly reduced tumor size (125). IL-15 gene therapy increased the proportion of CD4+ T cells and NK cells but had no effect on CD8+ T cells. Everolimus reduced the number of CD8+ T cells but had no effect on CD4+ T cells and NK cells compared to the control group. Although effective individually, no synergistic effect was observed with the combined treatment of everolimus and IL-15 gene therapy.

IL-10 is considered an immunosuppressive cytokine, which is related to various inhibitory and regulatory cell populations in the tumor microenvironment, including tolerogenic dendritic cells, regulatory CD4+ T cells, B10 cells, M2 macrophages, and myeloid-derived suppressor cells. Specific single nucleotide polymorphisms have been found to drive overexpression of the IL-10 gene, thereby promoting the progression of various malignant tumors. Multiple clinical studies have shown that upregulation of IL-10 levels is strongly correlated with tumor staging and poor prognosis in breast cancer patients (126). Shen et al. designed an IL-10 protein trap through genetic engineering methods and delivered it to the tumor microenvironment through lipid-arginine-DNA (LPD) nanoparticles to reduce IL-10 levels and activate dendritic cells, thereby enhancing the anti-tumor immune response (127). In the 4T1 triple-negative breast cancer model, IL-10 trap gene therapy alone could significantly inhibit tumor growth and improve the host’s survival time. The local and transient expression of the IL-10 trap gene can change the tumor microenvironment, reduce the accumulation of immunosuppressive cells, and promote the tumor-killing activity of cytotoxic T cells and NK cells. The potential of IL-10 as a therapeutic target is emphasized because it plays a key role in regulating the immunosuppressive microenvironment of breast cancer and other cancers.

Recombinant adenovirus-associated vector serotype 2 (rAAV2) is a viral vector widely used in gene therapy and gene function research. Yu et al. (128) constructed rAAV2-hIL15, which carries human interleukin 15 (hIL15) delivered to the body for long-term gene expression. The results showed that rAAV2-hIL15 can significantly delay the onset of breast cancer, inhibit tumor growth, and extend the lifespan of tumor-bearing mice. Moreover, rAAV2-hIL15 can express a large amount of IL15 protein, ultimately activating the cytotoxic activity of NK cells. This gene therapy strategy may be used to increase the local expression level of IL-15, thereby enhancing the activity of NK cells, and is expected to become a new potential therapeutic tool for immunotherapy of breast cancer.

## Clinical trials

In breast cancer treatment, several clinical trials have been conducted to explore the efficacy and safety of using NK cells or interleukins to influence NK cell activity. Burns et al. conducted a phase I/II clinical trial to evaluate the safety, immune activation effects, and potential efficacy of IL-2-activated NK cell infusion or IL-2 priming therapy in autologous transplantation for lymphoma and breast cancer (129). The results of this study indicated that IL-2-activated NK cell/IL-2 priming therapy could be safely administered, enhancing the cytolytic function of fresh peripheral blood mononuclear cells and increasing cytokine levels. However, with this dosage and IL-2 dosing regimen, no improvement in patient disease outcomes was observed. This study provided preliminary evidence for the potential therapeutic use of IL-2-activated NK cells post-autologous transplantation, although their clinical efficacy did not meet expectations, valuable information for future research and treatment strategies development. To enhance the therapeutic efficacy of IL-2-activated NK cells, exploring strategies such as increasing the number of NK cell infusions, using high-purity NK cells or their subsets, combining with tumor-reactive monoclonal antibodies, combination therapy with other cytokines like IL-12, or enhancing treatment efficacy through NK cell inhibitory receptor blockade may be necessary.

In a phase I clinical trial on Trastuzumab combined with IL-2 in HER2-positive metastatic breast cancer, it was found that Trastuzumab combined with IL-2 was a well-tolerated outpatient treatment regimen, resulting in NK cell expansion and enhanced *in vitro* targeted killing of HER2-expressing cells (130). This provided preliminary clinical trial results for the combination therapy of HER2-positive metastatic breast cancer, emphasizing the safety and potential clinical benefits of this treatment regimen. Recchia et al. conducted a clinical study on maintenance immunotherapy using IL-2 and 13-cis-retinoic acid (RA) in patients with metastatic breast cancer after chemotherapy (131). The study found that maintenance immunotherapy was well tolerated in patients with metastatic breast cancer and improved lymphocyte and NK cell counts and CD4+/CD8+ ratio, delaying disease recurrence. However, randomized trials are needed in the future to further validate these findings.

In a phase II clinical study evaluating allogeneic NK cell therapy in recurrent solid tumor patients (mainly ovarian cancer and breast cancer patients), lymphodepletion pre-treatment was administered to 20 patients, followed by infusion of NK cells from haploidentical donors and low-dose IL-2 to promote NK cell expansion. The infusion of allogeneic NK cells was associated with transient chimerism and may be limited by reconstructed recipient Treg cells (132). This study also mentioned some unforeseen serious adverse events, such as tumor lysis syndrome (TLS) and passenger lymphocyte syndrome (PLS), adding to the understanding of potential consequences of allogeneic NK cell therapy.

Recent phase I clinical trials have explored various administration methods of recombinant human IL-15 (rhIL-15), including intravenous infusion monotherapy, subcutaneous injection, and continuous intravenous infusion (133–135). Although these treatment methods significantly increase the total number of circulating NK cells, patients may experience adverse reactions related to recombinant cytokine administration.

These clinical studies will greatly impact the field of breast cancer treatment. We have listed relevant clinical studies in **Table 2**. It is worth noting that although interleukins theoretically have the potential to enhance immune responses and anti-tumor activity, their clinical application still faces challenges, including how to optimally activate the immune system, avoid excessive immune-related toxicity, and predict and select patient populations most likely to benefit from treatment.

**Table 2:** Clinical trial of NK Cell-Based therapy and the use of interleukins for the treatment of breast cancer and assessment of NK Cell status.

Treatment modalities	Phase	NCT	Status
Activation of primary NK cells	I/II	NCT03634501	UNKNOWN
Combination of allogeneic NK cells and targeted therapy	I	NCT05385705	RECRUITING
Combination of cryosurgery and NK cells immunotherapy	I/II	NCT02844335	COMPLETED
Combination of CAR-NK and targeted therapy	I	NCT05069935	TERMINATED
IL-7(observe the function of NK cells)	II	NCT01368107	COMPLETED
CAR-NK	I	NCT04106167	TERMINATED
Allogeneic NK cells as monotherapy and in combination with monoclonal antibody	I	NCT03319459	COMPLETED
Trastuzumab and IL-2(observe the function of NK cells)	II	NCT00006228	COMPLETED
Injection of rIL-2-activated NK cells	II	NCT00855452	COMPLETED

## Conclusion and outlook

NK cell-related therapies for breast cancer hold promise in altering the current landscape of breast cancer immunotherapy. A plethora of cytokines, including interleukins, can be utilized to broadly enhance the cytotoxicity and cytokine response of NK cells, and are currently undergoing clinical testing. These clinical trials leverage the unique characteristics of NK cells against breast cancer, adeptly executing tumor killing across various mechanisms. In addition to traditional approaches such as engineering NK cells, such as CAR-NK, or utilizing gene editing techniques to knock out inhibitory receptors on NK cells or inhibit immune checkpoints, modification of cytokines will also have profound effects on NK cell function. Previous modifications often targeted antibodies, such as developing bispecific or trispecific antibodies capable of simultaneously activating NK cells and binding to tumor antigens, enhancing ADCC effects, among others. Current research indicates that modifications can also be applied to cytokines, including interleukins. Utilizing engineered fusion complexes of interleukins and their receptors, encapsulated within extracellular vesicles, has been shown to retard tumor growth, increase tumor infiltration of NK cells and CD8+ T cells, elevate expression of IFN-γ, cytotoxic granule components, and anti-apoptotic BCL-2 (136). These studies hold strong promise for application and have entered clinical research stages.

However, there are still many limitations to NK cell-related therapies for breast cancer, and there is much room for developing novel approaches to further enhance the NK cell immunotherapy platform. Improving NK cell penetration and targeting in solid tumors, enhancing their activation, cytolytic capabilities, and survival rates after encountering the harsh immune-suppressive tumor microenvironment are the most critical goals. Additionally, efforts should be directed towards mobilizing exhausted tumor-infiltrating NK cells. These studies will profoundly change the landscape of breast cancer treatment, and we eagerly anticipate the arrival of this day.
